# Serum Neutralization Profiles of Straw-Colored Fruit Bats (*Eidolon helvum*) in Makurdi (Nigeria), against Four Lineages of Lagos Bat Lyssavirus

**DOI:** 10.3390/v13122378

**Published:** 2021-11-27

**Authors:** Veronica Odinya Ameh, Guanghui Wu, Hooman Goharriz, Rebecca Shipley, Anthony R. Fooks, Claude T. Sabeta, Lorraine M. McElhinney

**Affiliations:** 1 Department of Veterinary Public Health and Preventive Medicine, College of Veterinary Medicine, Federal University of Agriculture Makurdi, P.M.B., Makurdi 2373, Benue State, Nigeria; ameh.veronica@uam.edu.ng; 2 Department of Veterinary Tropical Diseases, Faculty of Veterinary Science, University of Pretoria, P Bag X04, Onderstepoort 0110, South Africa; SabetaC@arc.agric.za; 3 World Organisation for Animal Health (OIE), Rabies Reference Laboratory, Viral Zoonoses Group, Animal and Plant Health Agency (APHA), Weybridge, Woodham Lane, New Haw, Addlestone, Surrey KT15 3NB, UK; Hooman.Goharriz@apha.gov.uk (H.G.); Rebecca.shipley@apha.gov.uk (R.S.); tony.fooks@apha.gov.uk (A.R.F.); lorraine.mcelhinney@apha.gov.uk (L.M.M.); 4 World Organisation for Animal Health (OIE), Rabies Reference Laboratory, Agricultural Research Council-Onderstepoort Veterinary Research, Onderstepoort 0110, South Africa

**Keywords:** bats, lyssavirus, Lagos bat virus, *Eidolon helvum*, serology

## Abstract

Lagos bat lyssavirus (LBV) comprising four lineages (A, B, C and D) can potentially cause the fatal disease rabies. Although LBV-B was initially isolated in Nigeria in 1956, there is no information on LBV lineages circulating in Nigeria. This study was undertaken for the first time to measure the neutralizing antibodies against four lineages of LBVs in straw-colored fruit bats (*Eidolon helvum*) in Makurdi, Nigeria. Serum samples (*n* = 180) collected during two periods (November 2017–March 2018 and November 2018–March 2019) from terminally bled bats captured for human consumption were tested using a modified fluorescent antibody virus neutralization (mFAVN) assay. A high proportion of bat sera (74%) neutralized at least one lineage of LBV (with reciprocal titers from 9 to >420.89) and most of them neutralized LBV-A (63%), followed by LBV-D (49%), LBV-C (45%) and LBV-B (24%). The majority of positive sera (75%, *n* = 100) neutralized multiple LBV lineages while the remaining 25% (*n* = 33) neutralized only a single lineage, i.e., LBV-A (*n* = 23), LBV-D (*n* = 8) and LBV-C (*n* = 2). None exclusively neutralized LBV-B. The results suggest that exposure to LBV is common in *E. helvum* and that LBV-A (but not LBV-B) is likely to be circulating in this region of Nigeria.

## 1. Introduction

Lyssaviruses cause rabies, a fatal disease in animals and humans. Currently, there are 17 recognized lyssavirus species [1]. Lagos bat lyssaviruses (LBVs) have so far been exclusively identified in Africa. The first Lagos bat lyssavirus was isolated from a straw-colored fruit bat (*E. helvum*) on Lagos Island (Nigeria) in 1956 [2]. This lyssavirus was subsequently classified as a different lyssavirus species from classical rabies virus in 1970 based on results of complement fixation and virus neutralization tests and it was later classified as LBV lineage B (LBV-B) and remains the only LBV isolated from *E. helvum* in Nigeria so far [3,4,5,6]. Natural and experimental infections and pathogenesis of LBVs have been studied in *E. helvum* [7,8,9,10].

Currently, four lineages of LBV have been identified on the African continent. Lineage A (LBV-A) was isolated from *E. helvum* in Senegal, Ghana and Kenya and also from a *Rousettus aegyptiacus* bat exported to France from either Togo or Egypt [3,4,11]. No information on the distribution of lineage B (LBV-B) is available. Lineage C (LBV-C) was identified in *Epomophorus wahlbergi* in South Africa and *Micropteropus pussilus* on one occasion in the Central African Republic. In addition, LBV-C was confirmed in spill-over host species that included domestic cats (*Felis catus*) in South Africa and Zimbabwe, a water mongoose (*Atilax paludinosus*) in South Africa and a dog (*Canis familiaris*) in Ethiopia [3,4,5,12,13,14]. Lineage D (LBV-D) was identified in *Rousettus aegyptiacus* in Kenya and South Africa [5,13]. The four LBV lineages display the highest genetic diversity of all lyssavirus species based on comparisons of the complete nucleoprotein (N), phosphoprotein (P), matrix (M) and glycoprotein (G) genes and show some association with specific bat species [3,4,5].

*E. helvum* is a source of food for many human populations in various parts of Africa. Therefore, LBV can potentially pose a risk to the public, although no human infections have been associated with LBV. However, cases may have been missed because LBV infections cause the same clinical signs as rabies and the viruses can only be differentiated through antigenic and molecular typing techniques that are not widely performed in Africa. Vaccine protection data from laboratory studies suggest that the commercially available vaccines against rabies virus (a phylogroup I lyssavirus) provide little to no protection against lyssaviruses in phylogroup II, which consists of LBVs along with Mokola lyssavirus and Shimoni bat lyssavirus [15,16].

To infer the situation of LBV infection in *E. helvum* in Nigeria, this study was undertaken to test a panel of 180 serum samples collected from *E. helvum* in Makurdi, Benue State Nigeria against four lineages of LBV viruses (A–D).

## 2. Materials and Methods

Ethical approval for this research was granted by the Animal Ethics Committee and Research Ethics Committee of the University of Pretoria, with certificate numbers V092-18 (approval granted 26 November 2018) and REC097-18 (approval granted 6 February 2019). Approval to sample bat populations (VDS/194/S.4/11/T/85, on 28 November 2018) was also granted by the Director/Chief Veterinary Officer of Nigeria, Department of Veterinary and Pest Control Services, Federal Ministry of Agriculture and Pest Control Services, Abuja Nigeria. Serum samples were collected from terminally bled bats that were captured for human consumption in Makurdi, Benue State Nigeria (7°44′25.7″ N 8°31′52.8″ E). Bats were sampled from roosts on trees in two periods (November 2017–March 2018 and November 2018–March 2019) and identified morphologically. Sera were stored at −20 °C and then transported to the Animal and Plant Health Agency (UK), to test for the presence of neutralizing antibodies against the 4 lineages (A, B, C and D) of the Lagos bat lyssaviruses using a modified Fluorescent Antibody Virus Neutralization test (FAVN) [17,18].

A total of 180 serum samples were heat-inactivated at 56 °C for 30 min and then tested against LBV-A (RV767 = EF547432 = LBVAFR1999), LBV-B (RV1 = HM623779 = LBV.NIG56-RV1), LBV-C (RV134 = EF547425 = LBVSA1982) and LBV-D (RV3383 = KE476). Briefly, all serum samples were serially diluted 3-fold (1:3, 1:9, 1:27, 1:81 and 1:243 except for four samples that were performed up to 1:53,1441 against LBV-A to obtain the end-point titers) and were analyzed in duplicate. Each of the viruses (100 TCID_50_/50 µL) were incubated with 100 µL of the diluted serum at 37 °C for one hour for antibodies present in sera to neutralize the viruses. Baby Hamster Kidney fibroblast cells (BHK, 50 µL at 4.5–5 × 10^5^/mL) were added to the virus and serum mixtures and were incubated for 48 h at 37 °C with 5% CO_2_ to allow the growth of unneutralized viruses. The replicating viruses were detected after fixation and staining with fluorescein isothiocyanate (FITC)-conjugated anti-rabies N protein antibodies. The titer values were calculated using Spearman and Karber method and were expressed as the reciprocal of the dilution at which half of the wells had no replicating virus [18]. Positive and negative control plates were also prepared using positive LBV specific rabbit serum.

Antigenic cartography was used to quantitatively display the serological data as described previously [19,20]. Metric and ordinal multidimensional scaling techniques were used to optimize the relative positions of the virus and sera where neutralization occurred. Three-dimensional maps were used to visualize the antigenic data and determine antigenic relationships as the incremental increase in precision is negligible beyond three dimensions [21]. The two-dimensional maps were included for clarity.

The publicly available nucleotide sequences of the four lineages of glycoprotein (G) protein were downloaded from National Centre for Biotechnology Information (NCBI) nucleotide database (accessed on: 20 November 2020). They were either aligned directly using MagAlign version 15 program of DNASTAR Lasergene 15 or after being translated into the amino acid sequences using the EditSeq version 15 program within the DNASTAR Lasergene 15.

## 3. Results

The reciprocal titers of serum samples against four lineages of LBVs are shown in Table S1. The data are analyzed below.

### 3.1. Serum Samples Neutralized at Least One Lineage of LBV

A high number of bat sera (*n* = 133, 74%) neutralized at least one lineage of LBV (Table 1) using a reciprocal titer value of 9 as the cut-off. Even when a high cut-off value of 27 was used, half of the bat sera (52%) were still positive against at least one lineage of LBV viruses. The overlapping 95% confidence intervals show that the percentage differences between two different years or sexes of bats are not significant (Table 1).

**Figure 1:**
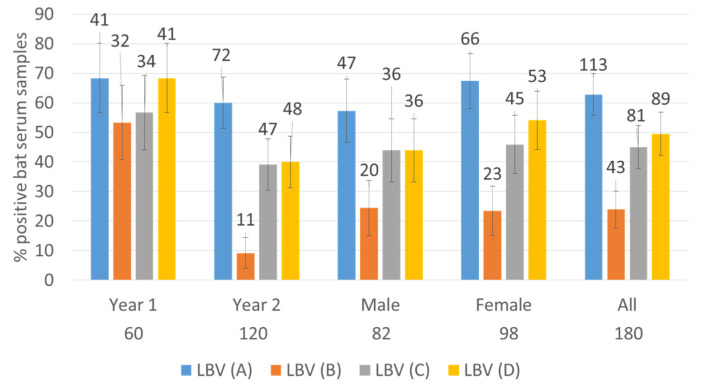
Distribution of positive serum samples against four lineages of LBV. The 95% confidence intervals of % are presented as error bars. The reciprocal titer value of 9 was used as the cut-off for this analysis. The number of positive samples are indicated over the bar and the sample numbers in each category are indicated below the graph.

The distribution of positive serum samples against four lineages of LBV is shown in Figure 1. Whilst the total percentages of bats positive for virus neutralizing antibodies (VNAs) in both years were not statistically different, the numbers of bats positive for VNAs were generally lower in the second year (i.e., November 2018–March 2019) with LBV-D and especially LBV-B being significantly lower (see the 95% confidence intervals, Figure 1).

### 3.2. Titer Distribution against Different Lineages of LBV

**Figure 2:**
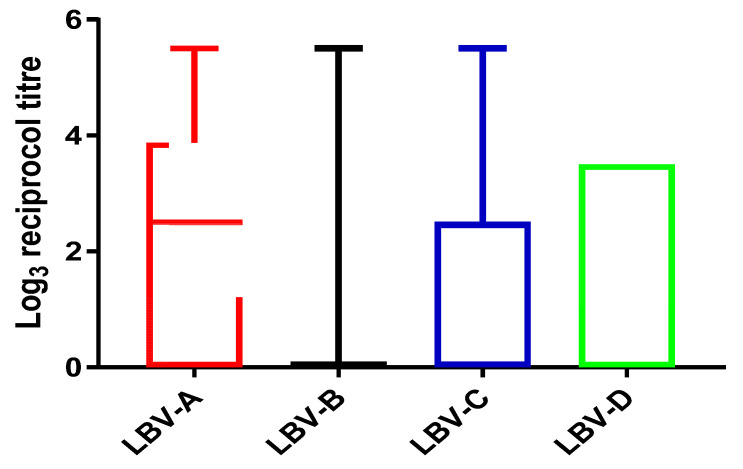
Box and whiskers plots display the log_3_ reciprocal titers against four lineages of LBV. Before log transformation, the samples that had no neutralization activity were given the value of one. The median log_3_ titer value for LBV-A is 2.5 (i.e., reciprocal value of 15.59) and 0 for the other three LBVs. The 75% percentiles are 3.9, 0, 2.5 and 3.5 for LBV-A, B, C and D respectively. The 25% percentiles are zero for all.

The titer values generated against all four lineages of LBV covered the full range (from 0 to ≥420.89, Figure 2) tested in this study. The median log_3_ titer values were only positive against LBV-A (log_3_15.59 = 2.5) and zeroes against all other three lineages. The 75% percentiles are log_3_ 70.6 = 3.9, log_3_1 = 0, log_3_ 15.59 = 2.5 and log_3_ 46.7 = 3.5 for LBV-A, B, C and D respectively. The 25% percentiles are zero for all.

Nine samples achieved ≥ 420.89 against LBV-A, one of which also had the same high titer against LBV-C, another against LBV-D, but seven of which had lower titers (or no titer) against other lineages of LBV. The only sample with a high titer against LBV-B (≥420.89) also had the same high titer against LBV-A and C. Five samples had high titer (≥420.89) against LBV-C, one of which had the same high titer (≥420.89) against LBV-A and B; another had the same high titer against LBV-D (≥420.89); three of which had lower titers against other lineages. Two samples achieved a high titer value (≥420.89) against LBV-D, one of which had the high titer value (≥420.89) against LBV-A and LBV-C and another shared the high titer value with LBV-A (Table S1). The numbers of positive samples against each LBV lineage were in the following order: LBV-A (*n* = 113, 63%, 95% CI of 56%–70%) > LBV-D (*n* = 89, 49%, 95% CI of 42% to 56%) > LBV-C (*n* = 81, 45%, 95%CI of 38% to 52%) > LBV-B (*n* = 43, 24%, 95% CI of 18% to 30%).

More samples achieved higher titers against LBV-A than against other LBV lineages.

The fewest samples neutralized LBV-B and most of those achieved relatively low titer values. Among positive serum samples, 50% of them had the highest titers against LBV-A (*n* = 67), 17% had highest titer against LBV D (*n* = 23), 7.5% had the highest titer against LBV C (*n* = 10) and only 0.75% had the highest titer against LBV-B (*n* = 1); this sample had a reciprocal titer of 27 against LBV-B, achieved titers of 15.95 against LBV-D and 9 against LBV-C). The difference between 27 and 15.95 is only one well, which is within the normal range of experimental error.

### 3.3. Serum Samples Neutralized Only One LBV Lineage

Both cut-off values (9 and 27) were used in the following analysis and the results are similar at both cut-offs (Table 2). The highest number of serum samples neutralized LBV-A. At the cut-off value of 27, one sample appeared to have exclusively neutralized LBV-B, but this sample achieved titers of 15.59 against LBV-D and 9 against LBV-C (see Section 3.2). Slightly more serum samples neutralized LBV-D and LBV-C (Table 2). It is interesting to note that even those samples with relatively high titer values against LBV-A (140.3) or LBV-C (81), or LBV-D (46.77 or 243) did not cross-neutralize with other lineages of LBV (i.e., titers were <9 or <27 depending on the cut-off). These data suggest that a proportion of the bats were infected with LBV-A, or possibly LBV-C or LBV-D or a related virus. The evidence does not support LBV-B infection amongst these bats as the neutralizing activity may be explained by cross-neutralization.

### 3.4. Serum Samples Neutralized Multiple Lineages of LBV

Among serum samples that displayed neutralizing activity against LBVs, the majority of them (75%, cut-off at 9 or 63%, cut-off at 27) neutralized more than one lineage of LBVs. Some serum samples neutralized two lineages of LBV (Table 3). More sera neutralized both LBV-A and LBV-D (n = 15 or 16) or LBV-A and LBV-C (*n* = 14 or 3) than those that neutralized LBV-B together with another lineage.

Among those serum samples that neutralized three lineages of LBV (Table 4), the numbers of sera that did not neutralize LBV-B were more than those that did not neutralize other LVB lineages.

### 3.5. *Antigenic Cartography*

**Figure 3:**
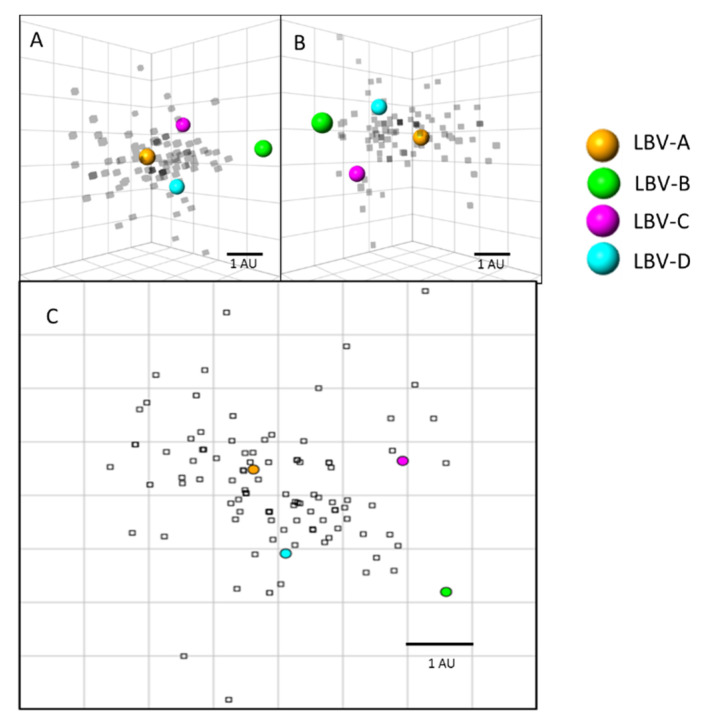
Antigenic cartography maps showing the antigenic distances of the sera tested in this study against the LBV lineages. (**A**) Three-dimensional antigenic map of LBVs and sera. Viruses (spheres) and sera (translucent-grey boxes) are positioned such that the distance from each serum to each virus is determined by the neutralization titer. Multidimensional scaling was used to position both sera and viruses relative to each other, so the orientation of the map within the axes is free. Scale bar shows one antigenic unit (AU). (**B**) The same antigenic map rotated to a different orientation for clarity. (**C**) Two-dimensional antigenic map based on the same data for clarity. The resolution of these antigenic maps in the average prediction error has been previously determined to be 1.22 (SE, 0.17) AU in 3D [21].

Antigenic cartography was used to visualize and quantify the antigenic relationships between the sera tested and the LBVs (Figure 3). Results showed that the majority of the sera positioned closest to LBV-A virus, followed by LBV-D, and LBV-C. LBV-B virus is positioned furthest from the other viruses and sera, reflecting less cross-neutralization of LBV-B by the sera tested. The extensive cross-neutralization between LBV-A, LBV-D, and LBV-C can be visualized on these maps as the majority of the sera are located antigenically closest to these viruses and some sera occupy a position on the map equidistant to these three LBV lineages. On average, all sera tested in this study positioned 1.3 antigenic units (AU) from LBV-A, 1.6 AU from LBV-D, 2.1 AU from LBV-C, and 3.1 AU from LBV-B.

### 3.6. Sequence Similarities among LBV Lineages

As a general principle, cross-neutralization occurs within the same phylogroup of lyssaviruses and LBV is just one species of lyssavirus within phylogroup II. Therefore, cross-neutralization among the four lineages of LBV should occur. The possible relationship between genetic distances and the (cross)-neutralization profiles observed in this study are explored below.

As neutralizing antibodies target the G protein, it is expected that similarities in G proteins may correlate with the cross-neutralization potential. To explore this, the available LBV G gene sequences were downloaded from NCBI nucleotide database and an alignment was performed (Figure S1). It shows that LBV-A was the most divergent from other lineages at the nucleotide level with LBV-C bifurcating into two branches. LBV-B sequences were closer with those of LBV-C and D.

As the neutralization activity is directed against antigens determined by amino acid sequences, an alignment of amino acid sequences should be more relevant. However, the alignment result (Figure S2) suggests that LBV-D is more distantly related to LBV-B and -C than LBV-A. It does not support the close relatedness of LBV-A and -D, and LBV-A and -C as suggested by the neutralizing profiles (Table 3 and Table 4).

The epitopes of LBV G proteins have not been mapped, but some regions of the rabies virus G protein were identified as binding sites for monoclonal antibodies. As all lyssavirus G proteins are similar in length (523 and 534 amino acids) and all contain 14 highly conserved cysteine residues alongside the antigenic domains that contribute to the structure of the protein [22], it is reasonable to expect that the corresponding regions of the LBV G proteins are also antigenic sites (Table 5). Based on this analysis, all LBV lineages share the same antigenic sites I, IV and “a”, but sites IIa, IIb and III contain different amino acid residues. LBV-C and LBV-Cv, a variant of LBV-C differed only at Site IIb. This shows that cross-neutralization can potentially happen among all lineages.

**Figure 4:**
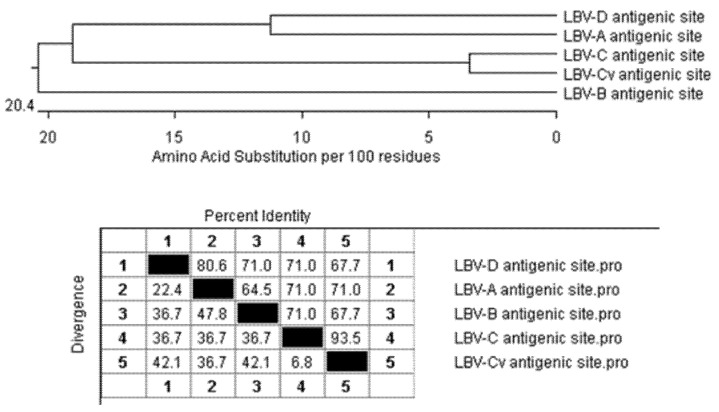
The alignment of concatenated antigenic site sequences (Table 5) using Clustal W in MagAlign version 15 of DNASTAR Lasergene 15.

When the sequences of these antigenic sites are concatenated and compared to each other (Figure 4), apart from LBV-C and its variant, the antigenic sites of LBV-A and LBV-D are most similar to each other with 80.6% identity. LBV-A is closer to LBV-C with 71% identity. LBV-B is the more distantly related to others.

## 4. Discussion

This work has demonstrated that a high number of bats had serum antibodies against at least one lineage of LBV, which suggests that those bats were infected by LBV or a related lyssavirus at some point in their lives and could pose a health hazard to the bat handlers and consumers.

This is the first attempt to address the question: which lineages of LBV may have been circulating in Nigeria? The new information will contribute to our understanding of the current status of LBV infections and the distribution of the LBVs and related viruses on the African continent.

### 4.1. Evidence Supports the Presence of LBV-A in Nigeria

Analyses showed that a high number (*n* = 113, 85% of the positive samples (*n* = 133), using the titer value of 9 as the cut-off) of sera had neutralizing activities against LBV-A. Many of them (*n* = 67, 50% of 133) generated higher titer values against LBV-A than against other LBV lineages (Figure 2 and Section 3.2). The results are graphically represented in Figure 3 where most serum samples have a closer distance to LBV-A than to other LBV lineages.

It would be reasonable to suggest that LBV-A is circulating among *E. helvum* in Nigeria based on the serological results presented here considering that LBV-A has been identified in *E. helvum* in countries nearby such as Ghana, Senegal and Kenya [3,4,11].

### 4.2. Serum Neutralization against LBV-B

In this study, only 43 (24%) bat sera neutralized LBV-B using the titer value 9 as the cut-off. This result is comparable with two previous studies where 19% of *E. helvum* serum samples from Northern Nigeria [23] and 37% from Ghana [24] were positive for VNAs against LBV-B. Another study found the 53% of *R. aegyptiacus* bats in Idanre area, Nigeria were seropositive against LBV-B [25]. Other LBV lineages were not tested in these three studies.

Importantly, no bat sera neutralized LBV-B exclusively and the serum titer values against LBV-B were not convincingly higher than those against other lineages (Section 3.2 and Section 3.3) and relatively fewer samples had neutralizing activity against LBV-B (Figure 1 and Figure 2, Table 2, Table 3 and Table 4 and Table S1). The results suggest that the neutralization was not specific to LBV-B, but more likely due to the cross-neutralization activities of antibodies generated against other lineages of LBV. Therefore, the seropositive rates against LBVs among those bats could have been underestimated in earlier studies. This is surprising as LBV-B was the first bat lyssavirus isolated from *E. helvum* on Lagos Island, Nigeria in 1956 [2]. *E. helvum* is distributed in wide areas of Africa, and they can migrate long distances, so the distance (600 km) between Makurdi and Lagos may not be the reason for the lack of LBV-B infection. The results suggest that LBV-B lineage may be no longer present in *E. helvum*. However, the high seropositive rate (53%) against LBV-B in *R. aegyptiacus* bats indicates the possible infection of LBV-B in *R. aegyptiacus* bats [25]. These bats were captured in Idanre, Nigeria about 200 km from Lagos, Nigeria from 2013 to 2020. Further studies are needed to understand the status of LBV-B in Nigeria and in different species of bats.

### 4.3. Serum Neutralization against LBV-C and LBV-D

A small number of serum samples neutralized LBV-D and LBV-C exclusively (Table 2). Nevertheless, some samples achieved higher titers against LBV-D (*n* = 23, 17%) or LBV-C (*n* = 10, 7.5%) than against other LBV lineages. From the neutralization profiles, it is possible to hypothesize that these bats may have been infected with LBV-C or -D if their sera only neutralized one of them or had the higher titer against one of them than against other lineages.

It is important to underscore that only LBV-A and -B have previously been isolated from *E. helvum.* LBV-C has never been identified in *E. helvum* and was mainly isolated in southern Africa, but was also detected in other animals in central Africa [3,4,5,12,13,14]. LBV-D has only been isolated from *Rousettus aegyptiacus* in Kenya and South Africa [5,13]. The results observed here could be due to cross-neutralization activities of antibodies generated against other lineages of LBV or other similar lyssaviruses that were not tested in this study. However, the possibility of exposure to LBV-C and LBV-D cannot be excluded. Further studies are warranted to investigate the infection of LBV-C and D among these bats.

### 4.4. Cross-Neutralization among LBV Lineages

Cross-neutralization among multiple lineages of LBVs or infections with multiple lineages of LBV may explain the results in Table 3 and Table 4. It is also possible that cross-neutralization would provide cross-protection making it unlikely for a bat to be infected by multiple lineages of LBV; although theoretically plausible, this concept remains highly improbable.

In an early study, Kuzmin and others [3] tested serum samples of *E. helvum* from Kenya against LBV-A, -B and -C, where three of four serum samples that neutralized LBV-A also neutralized LBV-B and-C. The authors attributed this to cross-neutralization. However, another study showed that sera from LBV-A-infected straw-colored fruit bats (*E. helvum*) from Ghana did not neutralize the LBV-B virus [11]. Therefore, cross-neutralization between LBV-A and LBV-B is not always present.

Markotter et al. pointed out that LBV-A was notably different from LBV-B and LBV-C isolates with the N gene sequence identity of around or less than 80%. The difference is sufficient to be classified as a new putative species, named Dakar bat lyssavirus (DBLV), since it was isolated from Dakar, Senegal [4].

The nucleotide sequence analysis of the G gene alignment (Figure S1) is consistent with LBV-A being the most divergent lineage based on the N gene sequences [4]. The alignment of amino acid sequences of the G protein (Figure S2) suggests that LBV-D is the most divergent. These alignments do not support the closer relatedness of LBV-A and -D, and LBV-A and –C in comparison with LBV-A and B as suggested by the neutralizing profiles (Table 3 and Table 4). The comparison of putative antigenic sites sequences provided a plausible explanation of the cross-neutralization profiles observed in this study (Figure 4) for the antigenic sites of LBV-A being more similar to LBV-D and C and less similar to LBV-B. Therefore, it can be deduced that the cross-neutralization between antibodies against LBV-B and other LBV lineages would be relatively low.

This explanation fits well with the results seen here, that the fewest number of serum samples neutralized lineage B while many co-neutralized lineages A and D or A and C (Table 2, Table 3 and Table 4). There is still much to learn about the antigenic properties of LBV G proteins and some antigenic sites may be more important than other sites. Furthermore, some epitopes are determined by protein conformation, which has not been considered here.

The complex neutralizing patterns observed in this study could be due to the fact that individual bats have generated different populations of antibodies as a result of varying affinity maturation even though they were infected by the same virus. The challenge dose and frequency of the challenge can also contribute to the diversity of the responses. Furthermore, the results may also be influenced by the presence of yet unidentified LBV lineages or similar viruses in Nigerian *E. helvum*.

### 4.5. Other Considerations

As no virus isolates or genetic sequences were obtained from the bat population, the neutralization results can only suggest the lineages of LBV that the bats were exposed to. Virus isolation should be performed to confirm the presence of LBV-A and other LBVs in bat species in Nigeria. In Nigeria, bats are usually associated with bad omens, witchcraft and supernatural powers. Therefore, it is less likely that individuals or the general public in this West African country would submit sick or dead bats to veterinary laboratories for testing. Public education and targeted surveillance are necessary to address this issue.

These bats were collected for consumption as food by local people as bats have become a special delicacy and a source of protein to people in some parts of Nigeria and West Africa. In Idanre Town in Ondo State, in Nigeria, people celebrate the Ilesun festival where bats are captured, cooked and eaten, and also sold in the markets. Hunters are sometimes bitten or scratched by bats which puts them in danger of being infected by lyssaviruses carried by bats [25]. The most frequently identified bat species roosting in the festival caves was *Rousettus aegyptiacus* species and ≥50% of them had neutralizing antibodies against phylogroup II lyssaviruses [25]. The current and previous studies suggest the frequent infection of phylogroup II lyssaviruses in Nigerian bats and the potential for humans to be infected. Bats are also known reservoirs of several viruses such as *Coronaviruses* [26,27,28], *Paramyxoviruses* [29,30,31], *Filoviruses* [32,33], *Influenza viruses* [34] and *Reoviruses* [35,36] and can serve as a source of zoonotic pathogens to their handlers and consumers.

Although no human infection of LBV was reported, the surveillance and diagnostic activities are limited in West Africa and the continent at large. The frequent human contact with infected bats can lead to cross-species transmission and the introduction of novel pathogens into the human population. It is necessary to enhance the surveillance and laboratory diagnostic capability in Africa so that infectious agents can be identified quickly. It is important to educate local populations to take appropriate precautions when coming into contact with bats and to understand the role of bats in maintaining a healthy ecosystem. It is essential to enhance the ability to report, respond and contain infectious disease outbreaks to protect public health and prevent any future pandemics.

## 5. Conclusions

High serum prevalence against LBV viruses was detected among *E. helvum* bats in Nigeria that were hunted as food by locals. These data suggest that many of these bats had been infected by LBV-A or related virus(es) rather than LBV-B, despite LBV-B being the only LBV lineage isolated from bats in Nigeria.

## Figures and Tables

**Table 1:** Positive serum antibodies against any lineage of LBV bat lyssaviruses.

	°% Positives (95% Confidence Intervals)									
Cut-Off	Both Years		Year 1		Year 2		Male		Female	
9	74	(67–80)	82	(72–91)	70	(62–78)	71	(61–81)	78	(69–86)
27	52	(44–59)	62	(49–74)	47	(38–56)	45	(34–56)	57	(47–67)
Total No.	180		120		60		82		98	

**Table 2:** Serum samples that neutralized only one lineage of LBV.

Cut Off	Lineages	A	B	C	D	Total
9	No. of sample	23	0	2	8	33
	Titer range	9–140.3	n/a	9	9–46.77	
27	No. of sample	24	1	3	7	35
	Titer range	27–140.3	27	27–81	27–243	

**Table 3:** Serum samples neutralized two lineages of LBV.

Cut-Off	Lineages	AB	AC	AD	BC	BD	CD	Total
9	No. of sample	4	14	15	0	1	4	38
	Titer range	9–140.3	9–420.89	15.95–140.3	n/a	15.99	9–140.3	
27	No. of sample	0	3	16	0	0	2	21
	Titer range	n/a	46.7–34,091.96	27–140.3	n/a	n/a	27–81	

**Table 4:** Serum samples neutralized 3–4 lineages of LBV viruses.

Cut-Off	Lineages	BCD (Not A)	ACD (Not B)	ABD (Not C)	ABC (Not D)	ABCD
9	No. of sample	5	24	1	1	31
	Highest titer	243	11,363.89	46.77	420.89	420.89
27	No. of sample	3	16	3	2	13
	Highest titer	46.7	11,363.99	140.3	420.89	729

**Table 5:** Antigenic sites on the LBV G proteins.

	Site IIb (34–42)	Site IIa (198–200)	Site I (226–231)	Site IV (263–264)	Site III (330–338)	Site ‘a’ (342–343)
LBV-A	GCSETSSFT	RKA	TLCGKP	NR	KRVDNWVDI	KG
LBV-B	GCGTSSVFS	KKS	TLCGKP	NR	LKVDNWSEI	KG
LBV-C	GCSDTATFS	KKS	TLCGKP	NR	LRVDSWNDI	KG
LBV-Cv	GCSNTATFN	KKS	TLCGKP	NR	LRVDSWNDI	KG
LBV-D	GCSTSTSFS	RKA	TLCGKP	NR	RRVDNWTDI	KG

## Data Availability

Data are contained within the article or supplementary material.

## Supplementary Materials

The following are available online at https://www.mdpi.com/article/10.3390/v13122378/s1, Figure S1: The alignment of nucleotide sequences of the glycoprotein genes (G) of four LBV lineages using Clustal W in MagAlign version 15 of DNASTAR Lasergene 15, Figure S2: The alignment of amino acid sequences of the glycoproteins of four LBV lineages using Clustal W in MagAlign version 15 of DNASTAR Lasergene 15, Table S1: Reciprocal titers of serum samples against each lineage of LBVs.
