# Supplementary material for: Serum Neutralization Profiles of Straw-Colored Fruit Bats (Eidolon helvum) in Makurdi (Nigeria), against Four Lineages of Lagos Bat Lyssavirus
Source: Viruses. 2021 Nov 27;13(12):2378. doi: 10.3390/v13122378 (PMC8706175; doi:10.3390/v13122378)
Supplement: Supplementary file 1 [file viruses-13-02378-s001.zip › viruses-1396313-supplementary.pdf]

Supplementary Materials

Table S1. Reciprocal titers of serum samples against each lineage of LBVs.

S/N	Sex	LBV A	LBV B	LBV C	LBV D
1	F	0.0	0.0	0.0	0.0
2	F	0.0	0.0	0.0	0.0
3	F	243.0	46.8	140.3	243.0
4	M	27.0	0.0	0.0	0.0
5	F	46.8	46.8	15.6	46.8
6	M	27.0	9.0	9.0	27.0
7	F	46.8	47.8	47.8	15.6
8	M	420.9	420.9	420.9	0.0
9	M	0.0	0.0	0.0	0.0
10	M	9.0	0.0	0.0	0.0
11	M	0.0	0.0	9.0	9.0
12	F	0.0	0.0	0.0	9.0
13	M	27.0	15.6	9.0	15.6
14	M	46.8	0.0	9.0	27.0
15	F	15.6	0.0	0.0	0.0
16	F	0.0	0.0	0.0	0.0
17	M	243.0	15.6	27.0	46.8
18	F	140.3	0.0	0.0	15.6
19	M	0.0	0.0	0.0	0.0
20	M	0.0	0.0	0.0	0.0
21	M	9.0	0.0	0.0	0.0
22	F	27.0	0.0	15.6	46.8
23	F	27.0	9.0	15.6	81.0
24	M	420.0	9.0	9.0	81.0
25	M	0.0	0.0	0.0	0.0
26	F	15.6	0.0	0.0	0.0
27	F	0.0	0.0	0.0	0.0
28	F	15.6	0.0	9.0	0.0
29	F	46.8	46.8	46.8	46.8
30	F	27.0	27.0	15.6	140.3
31	F	81.0	81.0	46.8	81.0
32	M	81.0	46.8	46.8	46.8
33	F	46.8	27.0	15.6	46.8
34	F	140.3	15.6	27.0	46.8
35	F	140.3	46.8	27.0	140.3
36	M	81.0	46.8	46.8	46.8
37	F	140.3	46.8	81.0	140.3
38	M	15.6	0.0	0.0	15.6
39	M	140.3	15.6	81.0	140.3
40	M	15.6	0.0	0.0	15.6
41	F	0.0	0.0	0.0	0.0
42	M	243.0	9.0	27.0	81.0
43	F	0.0	0.0	0.0	0.0
44	F	27.0	0.0	0.0	15.6
45	F	46.8	9.0	0.0	27.0
46	M	9.0	0.0	0.0	46.8
47	F	0.0	0.0	0.0	15.6

48	M	0.0	15.6	0.0	15.6
49	F	0.0	0.0	0.0	0.0
50	M	420.0	15.6	46.8	27.0
51	M	9.0	0.0	9.0	27.0
52	F	9.0	9.0	0.0	0.0
53	M	243.0	81.0	81.0	140.3
54	M	243.0	27.0	27.0	46.8
55	M	0.0	15.6	9.0	243.0
56	M	0.0	27.0	9.0	15.6
57	F	27.0	15.6	9.0	27.0
58	M	420.9	15.6	27.0	81.0
59	F	0.0	27.0	46.8	46.8
60	M	0.0	27.0	27.0	46.8
1	M	11364.0	0.0	27.0	46.8
2	M	140.3	0.0	15.6	0.0
3	F	0.0	0.0	0.0	0.0
4	F	9.0	0.0	0.0	0.0
5	F	0.0	0.0	0.0	0.0
6	F	140.3	140.3	46.8	46.8
7	F	0.0	0.0	0.0	0.0
8	M	81.0	0.0	420.9	243.0
9	M	0.0	0.0	0.0	0.0
10	F	15.6	0.0	15.6	0.0
11	F	0.0	0.0	9.0	0.0
12	F	46.8	0.0	0.0	0.0
13	F	46.8	0.0	420.9	0.0
14	F	0.0	0.0	81.0	27.0
15	F	0.0	0.0	0.0	0.0
16	M	0.0	0.0	9.0	0.0
17	M	0.0	0.0	0.0	0.0
18	F	0.0	0.0	9.0	140.3
19	F	0.0	0.0	0.0	0.0
20	F	0.0	0.0	0.0	0.0
21	F	15.6	0.0	0.0	15.6
22	M	15.6	0.0	0.0	15.6
23	F	15.6	0.0	46.8	0.0
24	F	9.0	0.0	0.0	0.0
25	F	15.6	0.0	81.0	0.0
26	F	9.0	0.0	0.0	0.0
27	F	27.0	0.0	0.0	15.6
28	M	15.6	0.0	0.0	0.0
29	F	34092.0	9.0	27.0	15.6
30	M	15.6	0.0	15.6	0.0
31	M	0.0	0.0	0.0	0.0
32	F	140.3	0.0	0.0	0.0
33	M	0.0	46.8	46.8	46.8
34	F	9.0	0.0	0.0	0.0
35	F	140.3	0.0	0.0	15.6
36	M	0.0	0.0	0.0	0.0
37	M	0.0	0.0	9.0	15.6
38	F	81.0	0.0	0.0	27.0

39	F	46.8	15.6	0.0	0.0
40	M	0.0	0.0	0.0	0.0
41	F	243.0	27.0	46.8	81.0
42	M	140.3	0.0	0.0	46.8
43	F	140.3	0.0	0.0	243.0
44	M	0.0	0.0	0.0	0.0
45	F	15.6	0.0	9.0	15.6
46	M	81.0	0.0	0.0	140.3
47	M	0.0	0.0	0.0	0.0
48	F	0.0	0.0	0.0	0.0
49	M	0.0	0.0	0.0	0.0
50	F	9.0	0.0	0.0	0.0
51	F	0.0	0.0	0.0	0.0
52	M	15.6	0.0	9.0	0.0
53	M	9.0	0.0	0.0	0.0
54	F	15.6	0.0	46.8	81.0
55	M	0.0	0.0	0.0	0.0
56	F	27.0	0.0	46.8	27.0
57	F	81.0	0.0	27.0	27.0
58	M	15.6	0.0	0.0	0.0
59	F	46.8	0.0	0.0	46.8
60	M	140.3	0.0	81.0	46.8
61	M	9.0	0.0	9.0	0.0
62	F	27.0	0.0	15.6	9.0
63	F	243.0	0.0	420.9	420.9
64	M	27.0	0.0	0.0	0.0
65	F	81.0	0.0	420.9	46.8
66	M	0.0	0.0	0.0	0.0
67	M	0.0	0.0	0.0	0.0
68	F	81.0	0.0	9.0	15.6
69	M	27.0	0.0	0.0	0.0
70	M	0.0	0.0	0.0	0.0
71	M	27.0	0.0	27.0	46.8
72	F	46.8	46.8	27.0	81.0
73	F	729.0	81.0	81.0	140.3
74	M	15.6	0.0	9.0	0.0
75	M	0.0	0.0	0.0	0.0
76	F	0.0	0.0	0.0	0.0
77	M	81.0	0.0	15.6	81.0
78	M	0.0	0.0	0.0	9.0
79	M	0.0	0.0	0.0	0.0
80	F	15.6	0.0	0.0	0.0
81	F	9.0	0.0	0.0	0.0
82	F	27.0	9.0	15.6	15.6
83	F	140.3	0.0	15.6	81.0
84	F	0.0	0.0	0.0	0.0
85	F	0.0	0.0	0.0	0.0
86	M	0.0	0.0	0.0	0.0
87	M	46.8	0.0	15.6	15.6
88	M	0.0	0.0	0.0	15.6
89	F	140.3	0.0	0.0	0.0

90	F	27.0	0.0	0.0	15.6
91	M	81.0	15.6	0.0	0.0
92	F	0.0	0.0	0.0	0.0
93	F	0.0	0.0	0.0	0.0
94	M	9.0	0.0	15.6	15.6
95	M	9.0	0.0	27.0	0.0
96	M	0.0	0.0	0.0	0.0
97	F	0.0	0.0	0.0	0.0
98	F	0.0	0.0	0.0	27.0
99	M	729.0	0.0	81.0	0.0
100	F	0.0	0.0	0.0	9.0
101	F	0.0	0.0	0.0	0.0
102	M	0.0	0.0	0.0	0.0
103	F	81.0	0.0	15.6	46.8
104	F	15.6	0.0	0.0	0.0
105	F	0.0	0.0	0.0	46.8
106	F	27.0	0.0	15.6	0.0
107	M	0.0	0.0	0.0	0.0
108	M	27.0	0.0	0.0	0.0
109	M	140.3	9.0	0.0	0.0
110	F	46.8	0.0	9.0	0.0
111	M	0.0	0.0	0.0	0.0
112	F	15.6	0.0	15.6	15.6
113	F	140.3	0.0	15.6	27.0
114	M	9.0	0.0	0.0	0.0
115	M	0.0	0.0	0.0	0.0
116	M	0.0	0.0	0.0	0.0
117	F	27.0	15.6	15.6	9.0
118	F	0.0	0.0	0.0	27.0
119	F	81.0	0.0	46.8	140.3
120	F	420.9	0.0	46.8	420.9

The first 60 samples were collected in year 1 and the rest (n=120) in year 2. Full range titrations (1:53,1441) were carried out on four samples against LBV-A.

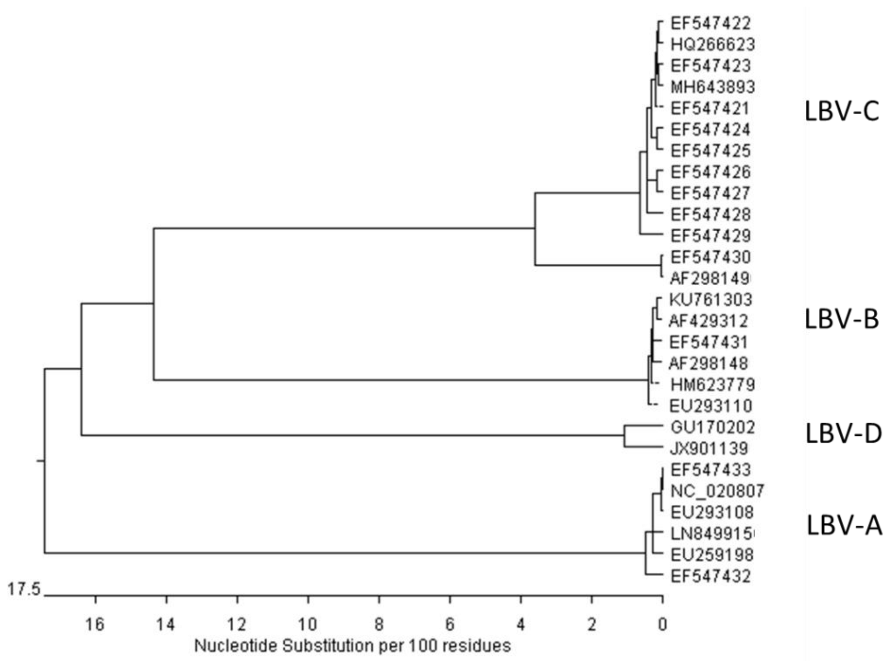

Figure S1. The alignment of nucleotide sequences of the glycoprotein genes (G) of four LBV lineages using Clustal W in MagAlign Version 15 of DNASTAR Lasergene 15.

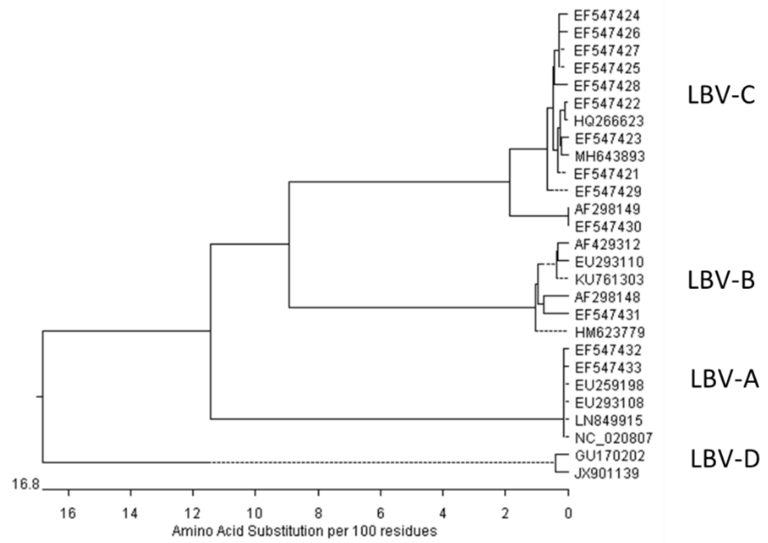

Figure S2. The alignment of amino acid sequences of the glycoproteins of four LBV lineages using Clustal W in MagAlign version 15 of DNASTAR Lasergene 15.
